# Assessment of Endothelial Dysfunction in Patients with Kawasaki Disease: A Meta-Analysis

**DOI:** 10.31083/j.rcm2308260

**Published:** 2022-07-20

**Authors:** Xiaona Yu, Dan Wu, Guang Song

**Affiliations:** ^1^Department of Ultrasound, Shengjing Hospital of China Medical University, 110004 Shenyang, Liaoning, China

**Keywords:** mucocutaneous lymph node syndrome, endothelial function, flow-mediated dilatation, biomarkers, meta-analysis

## Abstract

**Background:**

Kawasaki disease (KD) is a well-known systemic inflammatory vasculitis. Endothelial dysfunction is one of most easily overlooked non-coronary complications of KD. Several studies have assessed endothelial dysfunction using flow-mediated dilatation (FMD), nitroglycerin-mediated dilation (NMD), and biomarkers (E-selectin, P-selectin, intercellular adhesion molecule-1 (ICAM-1), and vascular cellular adhesion molecule-1 (VCAM-1)). However, the results were inconsistent and incomplete.

**Methods:**

We searched five databases for eligible studies until March 8, 2022. The summarized weighted mean difference (WMD) with 95% confidence intervals (CIs) were estimated for FMD, NMD, and four biomarkers level between KD and healthy children. A meta-analysis with subgroup analysis was conducted.

**Results:**

40 studies with a total of 2670 children (1665 KD patients and 1005 healthy children) were identified. During the acute phase, KD patients had lower FMD compared to the control group (WMD = –10.39, 95% CI: –13.80– –6.98). During the subacute phase, KD patients had lower FMD compared to the control group (WMD = –15.07, 95% CI: –17.61– –12.52). During the convalescence phase, KD patients had lower FMD and similar NMD compared to the control group (WMD = –4.95, 95% CI: –6.32– –3.58; WMD = –0.92, 95% CI: –2.39–0.55, respectively). During the convalescence phase, those KD patients without coronary artery lesion (CAL), with CAL, even with coronary artery aneurysm, had progressively lower FMD compared to healthy children (WMD = –3.82, 95% CI: –7.30– –0.34; WMD = –6.32, 95% CI: –7.60– –5.04; and WMD = –6.97, 95% CI: –7.99– –5.95, respectively). Compared to KD patients without CAL, those with CAL had lower FMD (WMD = –1.65, 95% CI: –2.92– –0.37). KD patients had higher levels of E-selectin, P-selectin, and ICAM-1 compared to healthy controls during different phases. KD patients had a higher level of VCAM-1 compared to healthy controls only during the acute phase (WMD = 61.62, 95% CI: 21.38–101.86).

**Conclusions:**

Endothelial dysfunction is present since the onset of KD and persists for years, confirmed by the measurement of FMD and biomarkers from different phases. An assumption is advanced that FMD impairment (the severity of endothelial dysfunction) may be positively correlated with CAL severity during the convalescence phase.

## 1. Introduction

Kawasaki disease (KD) is a well-known, self-limited, systemic inflammatory vasculitis, usually occurs in children between 6 months and 4 years of age. Among children in developed countries, KD has become the most common form of acquired cardiac disease [1]. The incidence of KD was estimated to be approximately 72–319 per 100,000 children in Asians, and 18–21 in the USA [2].

KD can lead to infiltration of inflammatory cells into small- and medium-sized arteries, particularly the coronary arteries. Up to 25% of KD patients can develop coronary artery aneurysm (CAA) unless they receive timely treatment with intravenous immunoglobulin [3]. Giant CAA could lead to coronary artery thrombosis, stenosis, or occlusion [4].

Endothelial dysfunction in KD by endothelial biomarkers (E-selectin, P-selectin, intercellular adhesion molecule-1 (ICAM-1), and vascular cellular adhesion molecule-1 (VCAM-1)) was identified as early in 1992 [5]. Afterwards the interest of investigation gradually increased from 1994–1999 [6, 7, 8, 9] to 2004–2018 [10, 11, 12, 13, 14, 15, 16, 17, 18, 19]. Endothelial dysfunction in KD by flow-mediated dilatation (FMD) and nitroglycerin-mediated dilation (NMD) stared in 1996 [20], since then the research of FMD continued to increase from 2001 to 2021 [21, 22, 23, 24, 25, 26, 27, 28, 29, 30, 31, 32, 33, 34, 35, 36, 37, 38, 39, 40, 41, 42, 43, 44]. More recently, Routhu *et al*. [43], performed assessment of endothelial dysfunction in acute and convalescent phases of KD using automated edge detection software. Wen *et al*. [44], also focused on the predictive value of brachial artery FMD on coronary artery abnormality in acute stage of KD.

Although the cause of endothelial dysfunction in KD remains unknown, endothelial activation and endothelial dysfunction are presumably due to increased cytokine production (e.g., IL-1, IL-6, and TNF) by immune effector cells via the NF-κB pathway [1, 45, 46]. The association between oxidative stress and endothelial dysfunction in early childhood KD patients was reported [41].

At the present, due to the absence of pathognomonic tests (a specific diagnostic test), the diagnosis continues to rest on the identification of principal clinical findings and the exclusion of other clinically similar entities with known causes. Therefore, FMD, NMD, and endothelial biomarkers will be instrumental in support to make KD diagnosis (in particular, in suspected atypical incomplete KD) [1], because of endothelial dysfunction as an early determinant of vascular disease [47]. In this review, we will discuss the emerging evidence demonstrating the clinical significance of FMD, NMD and endothelial biomarkers to KD during different (acute/subacute/convalescence) phases.

## 2. Materials and Methods

### 2.1 Protocol

This study was performed following a prospectively registered protocol in the PROSPERO database (CRD42022315266). This paper was reported in accordance with PRISMA guideline (**Supplementary Table 1**).

### 2.2 Study Identification and Selection

Two independent investigators (XY and DW) independently searched PubMed, MEDLINE, Embase, Cochrane library, and China National Knowledge Infrastructure (CNKI, China Core Journal Database) from database inception to March 8, 2022, to identify the relevant studies. The following search keywords included “Kawasaki disease” and (“flow-mediated dilatation”, “nitroglycerin-mediated dilation”, “E-selectin”, “P-selectin”, “intercellular adhesion molecule-1”, or “vascular cellular adhesion molecule-1”) [48, 49]. At the same time, we read the references of articles, trying to find potential literature which may meet the criteria.

Two researchers (XY and DW) independently screened the titles and abstracts for eligibility. Full papers were assessed to confirm disagreement in existence according to the exclusion criteria by the two researchers. Disagreements were discussed and resolved by involving a third reviewer (GS) for adjudication. Original studies were eligible if the following criteria were met: (i) observational study; (ii) the study investigated FMD/NMD or biomarkers in KD children compared to healthy participants; (iii) full text in English and Chinese available; (iv) the data of the acute phase must be acquired before intravenous immunoglobulin/aspirin treatment. Original studies were ineligible if the following criteria existed: (i) reviews, case reports, or case series; (ii) did not report the data necessary for calculating the mean and standard deviation of FMD/NMD or biomarkers level; (iii) animal studies; (iv) adult patients with a history of KD. If there were several publications from the same study, the study with the most cases and relevant information was included.

### 2.3 Data Extraction and Quality Assessment

The extracted data included the first author of involved studies, year of publication, country, groups, participant number, gender, mean age, time from KD onset, treatment, measurements of endothelial function, and whether the involved studies contain coronary artery lesion (CAL) group. Numeric data were gathered directly from tables or, when presented in graphs only, were inferred by digitizing the figure with GetData Graph Digitizer 2.26 [50]. The quality assessment was performed by the Newcastle–Ottawa Scale (NOS) assessment tool with the score 0–9. A score of 6 or more were considered to be high-quality studies.

### 2.4 Statistical Analysis

The pooled effects are presented as the weighted mean difference (WMD) with 95% confidence intervals (CIs). Heterogeneity was assessed using the *$I^{2}$* statistic. If there was no heterogeneity (*p *> 0.1 or *$I^{2}$* < 50%), a fixed-effects model was used to estimate the pooled effect; otherwise, a random-effects model was utilized. When heterogeneity existed, we conducted subgroup analyses. Sensitivity analyses were directed to assess the influence of the individual study on the overall estimate. We analyzed the symmetry of a funnel plot to evaluate possible small sample effects and used Begg’s and Egger’s tests to evaluate publication bias in the included studies. A *p-*value < 0.05 was considered statistically significant for asymmetry. Statistical analyses were performed using Stata (version 16.0; StataCorp, College Station, TX, USA).

## 3. Results

### 3.1 Study Selection and Characteristics of Eligible Studies

**Fig. 1.**
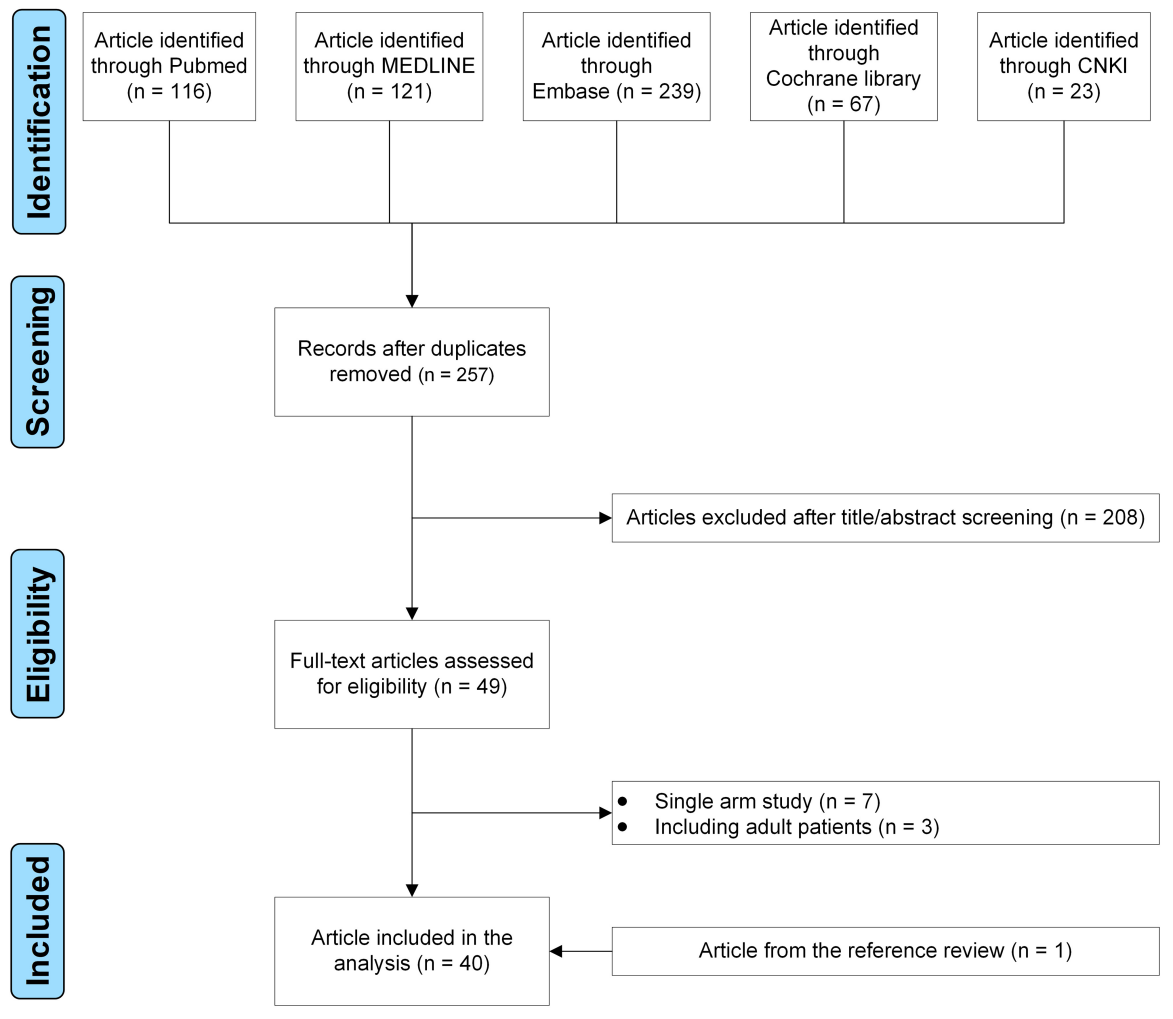
**The flow diagram of the study selection process**.

We had searched for potentially relevant publications from five sources. After applying the inclusion and exclusion criteria, 40 studies were identified (Fig. 1) [5, 6, 7, 8, 9, 10, 11, 12, 13, 14, 15, 16, 17, 18, 19, 20, 21, 22, 23, 24, 25, 26, 27, 28, 29, 30, 31, 32, 33, 34, 35, 36, 37, 38, 39, 40, 41, 42, 43, 44].

The baseline characteristics of the included studies are shown in Table 1 (Ref. [5, 6, 7, 8, 9, 10, 11, 12, 13, 14, 15, 16, 17, 18, 19, 20, 21, 22, 23, 24, 25, 26, 27, 28, 29, 30, 31, 32, 33, 34, 35, 36, 37, 38, 39, 40, 41, 42, 43, 44]). All studies were published between 1992 and 2021. Studies were conducted in Europe (Italy and UK), America (Canada and Chile), and Asia (China, India, Iran, Japan, Korea, and Turkey). 2670 children were included: 1665 children with KD and 1005 healthy participants. Twenty-five studies evaluated FMD, eight with NMD. Fifteen studies reported the difference of these four biomarkers between KD and control groups. Twenty of the involved studies contained CAL group. Quality assessment is shown in **Supplementary Table 2**. Details of flow-mediated dilation measurement in the involved studies are shown in **Supplementary Table 3**.

**Table 1.** **The baseline characteristics of the included studies**.

Study	Year	Country	Group	Number	Male/female	Mean age (year)	Time from KD onset	Treatment	Measurement	CAL subgroup	NOS score
Furukawa *et al*. [5]	1992	Japan	KD	29	15/14	1.8 ± 1.1	Acute phase: 2–9 days	29/29 received aspirin, 27/29 received IVIG	ICAM-1	No	8
							Convalescent phase: 20–194 days				
			Healthy control	10	6/4	2.00	–	–			
Kim *et al*. [6]	1994	Korea	KD	24	13/11	2.8 ± 2.1	Acute phase: 3–9 days	All patients received IVIG and aspirin	E-selectin	No	8
							Subacute phase: 15–42 days				
			Healthy control	10	NR	NR	–	–			
Nash *et al*. [7]	1995	UK	KD	59	32/27	2.5 ± 3.5	Acute phase: 0–28 days	NR	E-selectin, ICAM-1, VCAM-1	No	8
			Healthy control	48	37/11	4.9 ± 3.3	–	–			
Dhillon *et al*. [20]	1996	UK	KD	20	12/8	13.0 ± 2.0	Convalescent phase: 5.3–17.1 years	18/20 received aspirin, 3/20 received IVIG	FMD, NMD	No	7
			Healthy control	20	NR	15.0 ± 1.5	–	–			
Takeshita *et al*. [8]	1997	Japan	KD	16	7/9	1.6 ± 1.6	Acute phase: 3–7 days	All patients received IVIG and aspirin	E-selectin, P-selectin, VCAM-1	No	8
							Subacute phase: 11–19 days				
							Convalescent phase: 28–41 days				
			Healthy control	10	6/4	3.9 ± 1.5	–	–			
Schiller *et al*. [9]	1999	Italy	KD	30	20/10	2.8 ± 3.3	Acute phase: before treatment	All patients received IVIG and aspirin	E-selectin, ICAM-1	No	8
							Subacute phase: 1–2 days after treatment				
							Convalescent phase: 42–90 days				
			Healthy control	15	9/6	7.1 ± 3.3	–	–			
Silva *et al*. [21]	2001	Canada	KD	24	18/6	14.3 ± 1.8	Convalescent phase: 11.3 ± 1.8 years	23/24 received aspirin, 14/24 received IVIG	FMD, NMD	No	8
			Healthy control	11	6/5	14.1 ± 1.5	–	–			
Deng *et al*. [22]	2002	China	KD	39	28/11	7.1 ± 2.7	Convalescent phase: 1.0–10.0 years	All patients received aspirin, 34/39 received IVIG	FMD, NMD	Yes	9
			Healthy control	17	13/4	7.0 ± 3.1	–	–			
Qiu *et al*. [10]	2004	China	KD	36	21/15	1–4	Acute phase: 3–7 days	All patients received IVIG and aspirin	E-selectin, P-selectin	Yes	9
							Subacute phase: 11–19 days				
							Convalescent phase: 28–41 days				
			Healthy control	30	NR	NR	–	–			
Kadono *et al*. [23]	2005	Japan	KD	24	13/11	8.3 ± 4.1	Convalescent phase: 5.8 ± 4.6 years	2/24 received aspirin	FMD	Yes	8
			Healthy control	41	29/12	10.7 ± 4.4	–	–			
Sun_1 *et al*. [24]	2005	China	KD	22	16/6	5.3 ± 4.0	Convalescent phase: 1.8 ± 1.9 years	NR	FMD	Yes	7
			Healthy control	16	11/5	5.5 ± 4.1	–	–			
Sun_2 *et al*. [25]	2005	China	KD	36	24/12	2.9 ± 1.9	Acute phase: less than one month	NR	FMD	No	6
			Healthy control	15	9/6	3.2 ± 1.9	–	–			
Zhang *et al*. [11]	2005	China	KD	26	20/6	1.8 ± 2.9	Acute phase: before treatment	All patients received IVIG	E-selectin	Yes	7
							Subacute phase: 3 days after treatment				
			Healthy control	15	9/6	2.1 ± 0.5	–	–			
Wang *et al*. [12]	2006	China	KD	20	11/9	0.8–5	Acute phase: before treatment	All patients received IVIG and aspirin	E-selectin, ICAM-1	No	6
							Subacute phase: 1–3 days after treatment				
			Healthy control	19	11/8	0.9–5	–	–			
Li *et al*. [13]	2007	China	KD	34	23/11	1.1 ± 1.6	Acute phase: before treatment	All patients received IVIG and aspirin	VCAM-1	No	7
							Convalescent phase: NR				
			Healthy control	26	14/12	1.4 ± 1.7	–	–			
Liu *et al*. [26]	2007	China	KD	101	78/33	6.8 ± 2.3	Convalescent phase: 4.4 ± 1.9 years	NR	FMD	Yes	8
			Healthy control	103	64/39	7.2 ± 2.5	–	–			
McCrindle *et al*. [27]	2007	Canada	KD	52	35/17	15.5 ± 2.3	Convalescent phase: 11.2 ± 3.7 years	48/52 received aspirin, 33/52 received IVIG	FMD	Yes	9
			Healthy control	60	30/30	14.9 ± 2.4	–	–			
Borzutzky *et al*. [28]	2008	Chile	KD	11	7/4	10.6 ± 2.0	Convalescent phase: 8.1 ± 3.6 years	All patients received IVIG and aspirin	FMD	No	8
			Healthy control	11	7/4	10.4 ± 1.8	–	–			
Huang *et al*. [29]	2008	China	KD	11	8/3	12.9 ± 2.5	Convalescent phase: 10.8 ± 3.0 years	All patients received IVIG and aspirin	FMD	Yes	9
			Healthy control	11	8/3	13.0 ± 2.4	–	–			
Xu_1 *et al*. [14]	2008	China	KD	40	27/13	2.3 ± 3.4	Acute phase: before treatment	NR	VCAM-1	No	6
							Subacute phase: 5–7 days after treatment				
			Healthy control	30	NR	NR	–	–			
Xu_2 *et al*. [15]	2008	China	KD	40	27/13	2.3 ± 3.4	Acute phase: before treatment	NR	P-selectin	No	6
							Subacute phase: 5–7 days after treatment				
			Healthy control	30	NR	NR	–	–			
Ghelani *et al*. [30]	2009	India	KD	20	13/7	8.4 ± 2.3	Convalescent phase: 0.25–6.5 months	All patients received IVIG and aspirin	FMD	No	8
			Healthy control	20	13/7	8.6 ± 2.6	–	–			
Liu *et al*. [31]	2009	China	KD	41	25/16	7.1 ± 1.8	Convalescent phase: 1.5–10 years	21/41 received aspirin, 41/41 received IVIG	FMD	Yes	9
			Healthy control	22	13/9	8.4 ± 2.7	–	–			
Chen *et al*. [16]	2010	China	KD	148	98/50	2.2 ± 1.8	Acute phase: 3–7 days	All patients received IVIG and aspirin	E-selectin	Yes	9
							Subacute phase: 11–19 days				
							Convalescent phase: 28–41 days				
			Healthy control	20	14/6	2.0 ± 1.7	–	–			
Straface *et al*. [17]	2010	Italy	KD	12	NR	0.5–2	Acute phase: before treatment	All patients received IVIG and aspirin	P-selectin	No	8
			Healthy control	5	NR	NR	–	–			
Duan *et al*. [32]	2011	China	KD	31	22/9	6.2 ± 3.4	Convalescent phase: 1–12.5 years	NR	FMD, NMD	Yes	8
			Healthy control	21	14/7	5.7 ± 2.5	–	–			
Liu *et al*. [18]	2013	China	KD	271	182/89	2.9 ± 2.2	Acute phase: before treatment	All patients received IVIG	ICAM-1	No	8
							Subacute phase: 1–2 days after treatment				
			Healthy control	36	21/15	3.2 ± 1.7	–	–			
Ishikawa *et al*. [33]	2013	Japan	KD	24	14/10	6.5 ± 1.8	Convalescent phase: 1–4.1 years	4/24 received aspirin, 24/24 received IVIG	FMD, NMD	Yes	9
			Healthy control	22	13/9	7.9 ± 2.8	–	–			
Ding *et al*. [34]	2014	China	KD	28	16/12	1.9 ± 1.8	Acute phase: 1–11 days	All patients received IVIG and aspirin	FMD	No	8
							Subacute phase: 11–21 days				
							Convalescent phase: >1 month				
			Healthy control	28	16/12	1.8 ± 1.8	–	–			
Duan *et al*. [35]	2014	China	KD	13	13/0	5.8 ± 2.1	Convalescent phase: 1.5–7 years	All patients received IVIG and aspirin	FMD, NMD	Yes	9
			Healthy control	14	14/0	5.5 ± 2.3	–	–			
Laurito *et al*. [36]	2014	Italy	KD	14	9/5	10.0 ± 3.7	Convalescent phase: 6.3 ± 4.8 years	NR	FMD	Yes	8
			Healthy control	14	7/7	10.2 ± 2.4	–	–			
Gao *et al*. [37]	2015	China	KD	50	35/15	2.0 ± 2.2	Acute phase:1–11 days	NR	FMD	Yes	9
							Subacute phase: 11–21 days				
							Convalescent phase: >1 month				
			Healthy control	19	11/8	2.0 ± 3.0	–	–			
Sabri *et al*. [38]	2015	Iran	KD	16	7/9	12.1 ± 5.0	Convalescent phase: 5.8 ± 3.7 years	NR	FMD	No	7
			Healthy control	19	10/9	12.6 ± 4.5	–	–			
Mori *et al*. [39]	2016	Japan	KD	67	36/31	9.5 ± 2.5	Convalescent phase: 7.5 ± 2.4 years	NR	FMD	Yes	7
			Healthy control	28	16/12	8.6 ± 2.2	–	–			
Parihar *et al*. [40]	2017	India	KD	20	12/8	11.5 ± 3.7	Convalescent phase: 4.5 ± 1.9 years	All patients received IVIG and aspirin	FMD	Yes	7
			Healthy control	20	12/8	11.5 ± 3.3	–	–			
Ishikawa *et al*. [41]	2018	Japan	KD	25	12/13	7.0 ± 3.7	Convalescent phase: 4.1 ± 1.5 years	All patients received IVIG and aspirin	FMD, NMD	Yes	9
			Healthy control	25	12/13	6.4 ± 2.7	–	–			
Pi* et al*. [19]	2018	China	KD	44	29/15	2.6 ± 2.1	Acute phase: before treatment	All patients received IVIG and aspirin	P-selectin	Yes	8
							Subacute phase: 7–14 days after treatment				
			Healthy control	23	16/7	2.4 ± 1.5	–	–			
Cetiner *et al*. [42]	2021	Turkey	KD	26	21/5	8.2 ± 3.8	Convalescent phase: 1–15 years	All patients received IVIG	FMD	No	7
			Healthy control	26	22/4	9.0 ± 3.4	–	–			
Routhu *et al*. [43]	2021	India	KD	16	12/4	4.0 ± 4.0	Acute phase: 1–15 days	All patients received IVIG and aspirin	FMD	No	8
							Convalescent phase: 3–12 months				
			Healthy control	16	10/6	5.1 ± 2.3	–	–			
Wen *et al*. [44]	2021	China	KD	105	62/43	2.9 ± 1.9	Acute phase: before treatment	All patients received IVIG and aspirin	FMD	Yes	9
			Healthy control	79	45/34	3.2 ± 1.8	–	–			

CAL, coronary artery lesion; FMD, flow-mediated dilatation; ICAM-1, intercellular adhesion molecule-1; IVIG, intravenous immunoglobulin; KD, Kawasaki disease; NMD, nitroglycerin-mediated dilation; NOS, New castle Ottawa Scale; NR, no reported; VCAM-1, vascular cellular adhesion molecule-1.

### 3.2 Difference of FMD/NMD between KD and Healthy Groups, and Subgroup Analysis

**Fig. 2.**
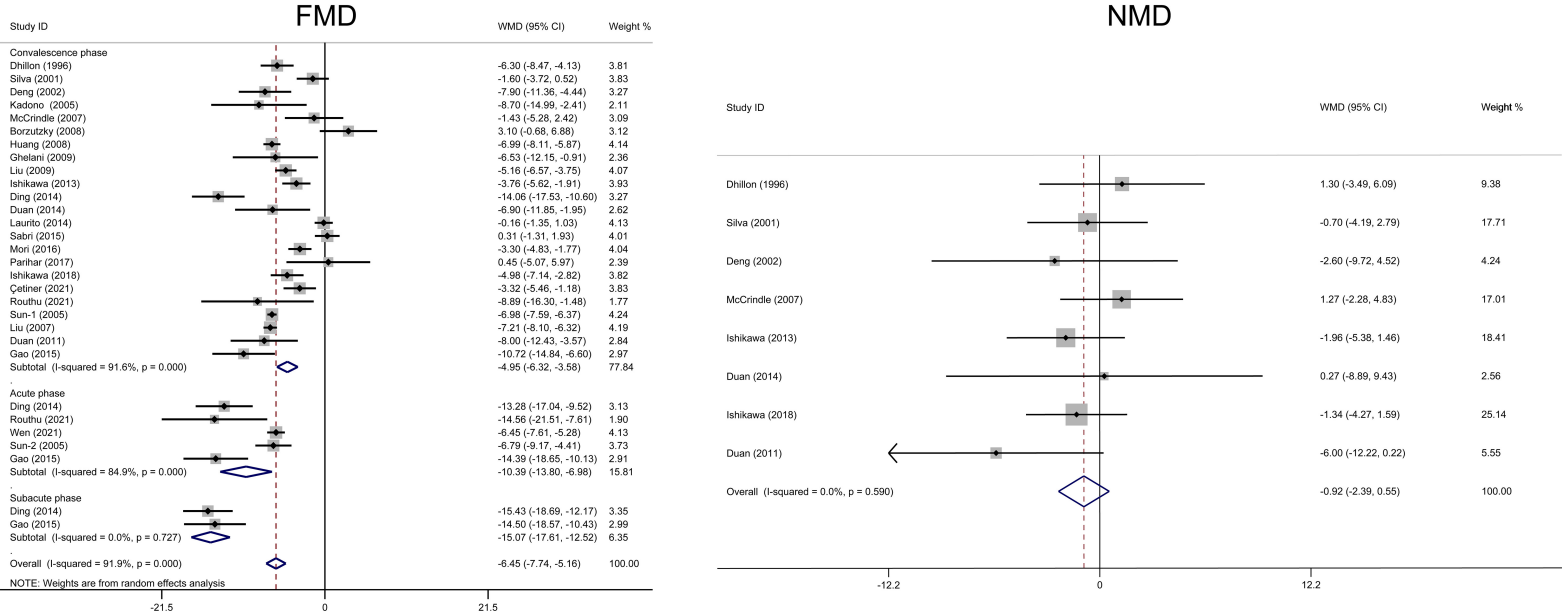
**Forest plots of the meta-analysis of FMD/NMD between Kawasaki disease and healthy children during different phases**. CI, confidence interval; FMD, flow-mediated dilatation; NMD, nitroglycerin-mediated dilation; WMD, weighted mean difference.

Five studies with 235 KD and 157 healthy children assessed FMD in the acute phase [25, 34, 37, 43, 44]. During this phase, KD patients had lower FMD compared to the control group (WMD = –10.39, 95% CI: –13.80– –6.98, *p *< 0.001, *$I^{2}$* = 84.9%, Fig. 2). No possible source of this heterogeneity was found after subgroup analysis. Subgroup analyses indicated that significant differences were observed in most subgroup analyses (Table 2).

**Table 2.** **Subgroup analyses of FMD between Kawasaki disease and healthy control**.

		Number of studies	Test of difference		Test of heterogeneity	
			WMD (95% CI)	*p* value	*$I^{2}$* (%)	*p* value
FMD (%) in the acute phase						
Language						
	English	3	–10.89 (–16.72– –5.07)	<0.001	87.5	<0.001
	Chinese	2	–10.38 (–17.81– –2.94)	0.006	89.3	0.002
Country						
	China	4	–9.76 (–13.29– –6.23)	<0.001	86.7	<0.001
	India	1	–14.56 (–21.51– –7.61)	<0.001	-	-
Occlusion position						
	Foream	2	–9.96 (–17.45– –2.48)	0.009	76.7	0.038
	Others	3	–11.12 (–16.92– –5.31)	<0.001	91	<0.001
Occlusion’ pressure						
	50 mmHg above resting SBP	4	–9.76 (–13.29– –6.23)	<0.001	86.7	<0.001
	250 mmHg	1	–14.56 (–21.51– –7.61)	<0.001	-	-
FMD (%) in the convalescence phase						
Language						
	English	19	–4.28 (–5.88– –2.68)	<0.001	89.3	<0.001
	Chinese	4	–7.15 (–7.73– –6.57)	<0.001	9.6	0.345
Country						
	Europe	2	–3.16 (–9.18–2.86)	0.303	95.8	<0.001
	America	3	–0.22 (–3.04–2.61)	0.881	57.6	0.095
	Asia	18	–5.92 (–7.22– –4.62)	<0.001	87.7	<0.001
Follow-up duration						
	>1 year	15	–4.67 (–6.00– –3.34)	<0.001	81.3	<0.001
	<1 year	1	–8.89 (–16.30– –1.48)	0.019	-	-
	Mixed	7	–5.62 (–8.97– –2.27)	0.001	96.7	<0.001
Occlusion position						
	Foream	14	–5.13 (–6.80– –3.46)	<0.001	93.2	<0.001
	Upper arm	3	–5.00 (–9.75– –0.26)	0.039	83.7	0.002
	NR	6	–4.39 (–8.30– –0.47)	0.028	90.2	<0.001
Occlusion’ pressure						
	50 mmHg above resting SBP	7	–5.57 (–8.93– –2.21)	0.001	90.6	<0.001
	200 mmHg	8	–6.02 (–7.29– –4.75)	<0.001	59.1	0.017
	Others	8	–3.20 (–5.65– –0.75)	0.011	93	<0.001
Occlusion duration						
	5 mins	20	–5.18 (–6.71– –3.65)	<0.001	92.1	<0.001
	Others	3	–3.71 (–6.13– –1.28)	0.003	79	0.009

CI, confidence interval; FMD, flow-mediated dilatation; NR, no reported; SBP, systolic blood pressure; WMD, weighted mean difference.

Only two studies with 75 KD and 47 healthy children assessed FMD in the subacute phase [34, 37]. During this phase, KD patients had lower FMD compared to the control group (WMD = –15.07, 95% CI: –17.61– –12.52, *p *< 0.001, *$I^{2}$* = 0%, Fig. 2).

Twenty-three studies with 693 KD children and 584 healthy participants assessed FMD in the convalescence phase. During this phase, KD patients had lower FMD compared to the control group (WMD = –4.95, 95% CI: –6.32– –3.58, *p *< 0.001, *$I^{2}$* = 91.6%, Fig. 2). No possible source of this heterogeneity was found after subgroup analysis. Subgroup analyses indicated that significant differences were observed in most subgroup analyses (Table 2).

Eight studies with 228 KD children and 190 healthy participants assessed NMD in the convalescence phase. Sublingual nitroglycerin (glyceryl trinitrate) was administrated before NMD measurement in all these eight studies. During this phase, KD patients had similar NMD compared to the control group (WMD = –0.92, 95% CI: –2.39–0.55, *p* = 0.219, *$I^{2}$* = 0%, Fig. 2).

### 3.3 Association of FMD Measures with Severity of Coronary Artery

Only one study in the acute phase [44] and none in the subacute phase assessed the association of FMD measures with the severity of CAL.

**Fig. 3.**
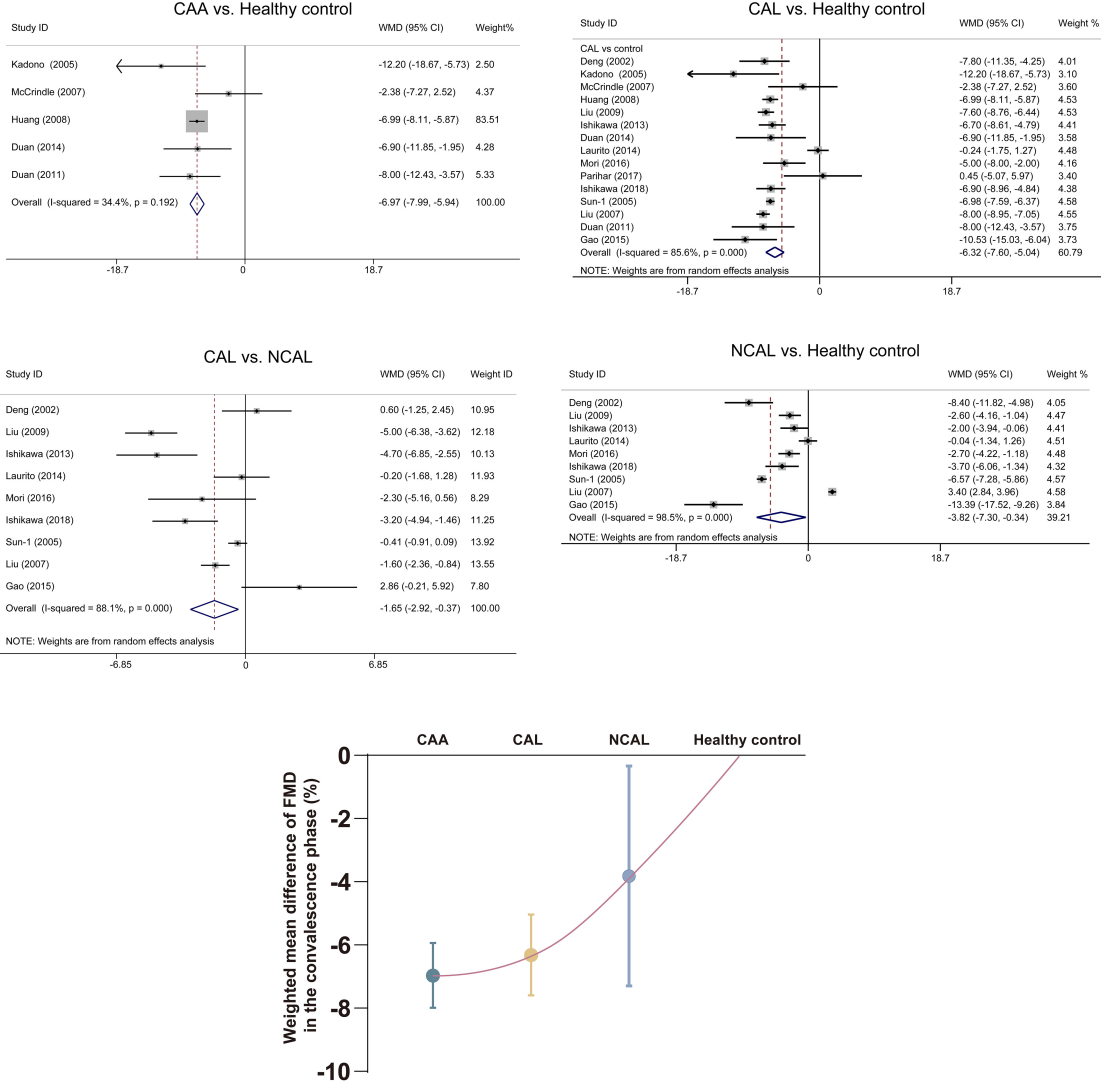
**Association of FMD measures with severity of coronary artery in the convalescence phase**. Data are expressed as mean with 95% CI. CAA, coronary artery aneurysm; CAL, coronary artery lesion; CI, confidence interval; FMD, flow-mediated dilatation; NCAL, no coronary artery lesion; WMD, weighted mean difference.

During the convalescence phase, those KD patients without CAL, with CAL, even with CAA, had progressive lower FMD compared to healthy children (WMD = –3.82, 95% CI: –7.30– –0.34; WMD = –6.32, 95% CI: –7.60– –5.04; and WMD = –6.97, 95% CI: –7.99– –5.95, respectively; all *p *< 0.05; Fig. 3). Compared to KD patients without CAL, those with CAL had lower FMD (WMD = –1.65, 95% CI: –2.92– –0.37, Fig. 3). Therefore, a positive correlation seems to exist between FMD impairment and CAL severity in KD patients during the convalescence phase (Fig. 3).

### 3.4 Difference of Biomarkers between KD and Healthy Groups

**Fig. 4.**
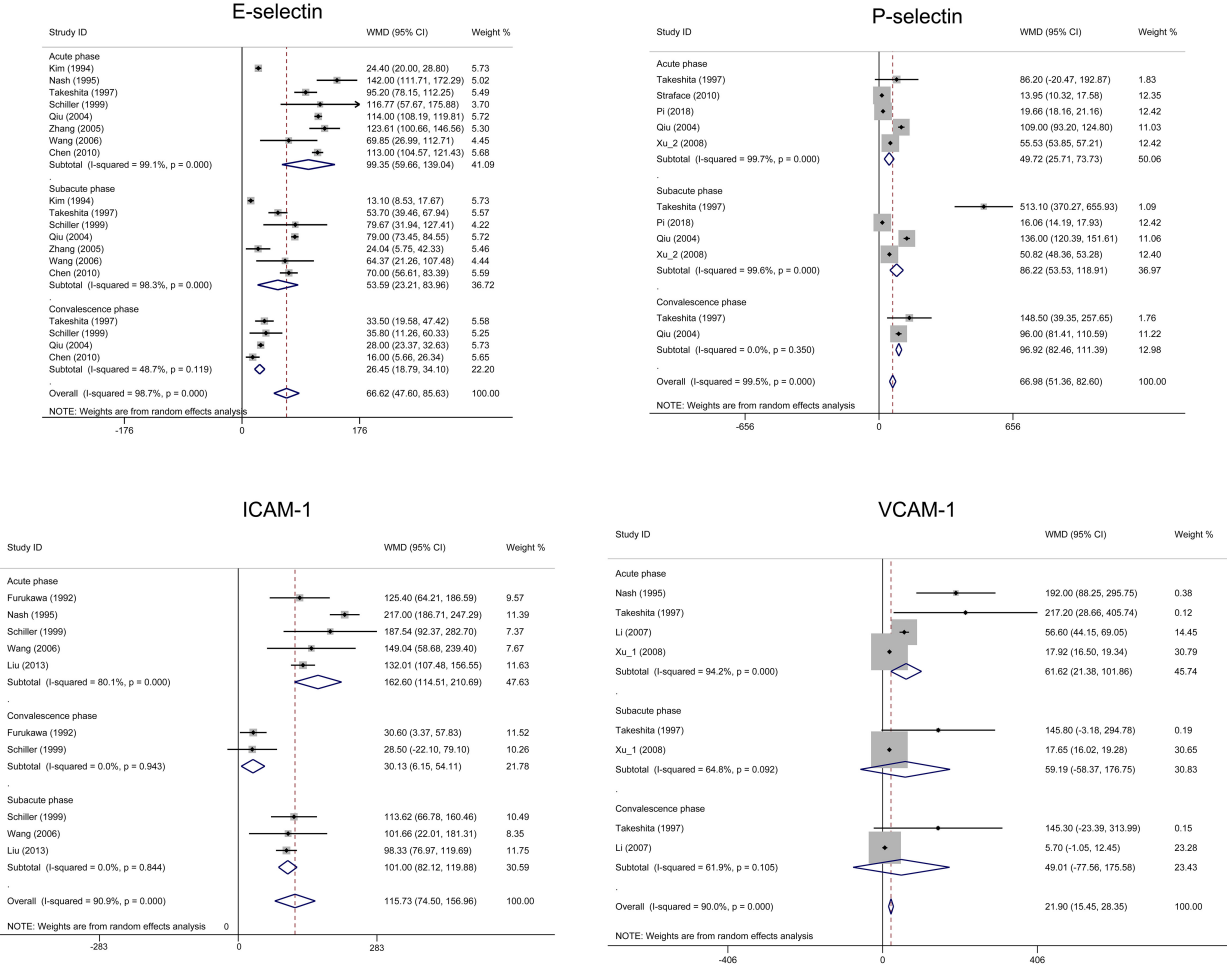
**Forest plots of the meta-analysis of four biomarkers between Kawasaki disease and healthy children during different phases**. CI, confidence interval; ICAM, intercellular adhesion molecule-1; VCAM, vascular cellular adhesion molecule-1; WMD, weighted mean difference.

KD patients had higher levels of E-selectin, P-selectin, and ICAM-1 compared to the healthy control during different phases (Fig. 4). KD patients had a higher level of VCAM-1 compared to the healthy control only during the acute phase (WMD = 61.62, 95% CI: 21.38–101.86). There was no difference in VCAM-1 between KD and the healthy control group during the subacute and convalescence phases.

### 3.5 Sensitivity Analysis and Publication Bias

To evaluate the robustness of the results, sensitivity analyses were performed by sequentially removing each study. No apparent change occurred for most outcomes when an individual study was omitted.

No publication bias was observed in our evaluation of the funnel plots for FMD (convalescence phase), NMD, and four biomarkers, confirmed by Begg’s and Egger’s tests (**Supplementary Table 4**, **Supplementary Figs. 1–3**). However, an obvious publication bias was revealed in our evaluation of the funnel plots for FMD (during acute phase), confirmed by Egger’s (*p* = 0.038) tests; Thus, the trim-and-fill method was used to adjust the publication bias. After trimming, the results were similar, indicating that the results were statistically reliable (WMD = 0.84, 95% CI: 0.66–1.02, **Supplementary Fig. 4**).

## 4. Discussion

**Fig. 5.**
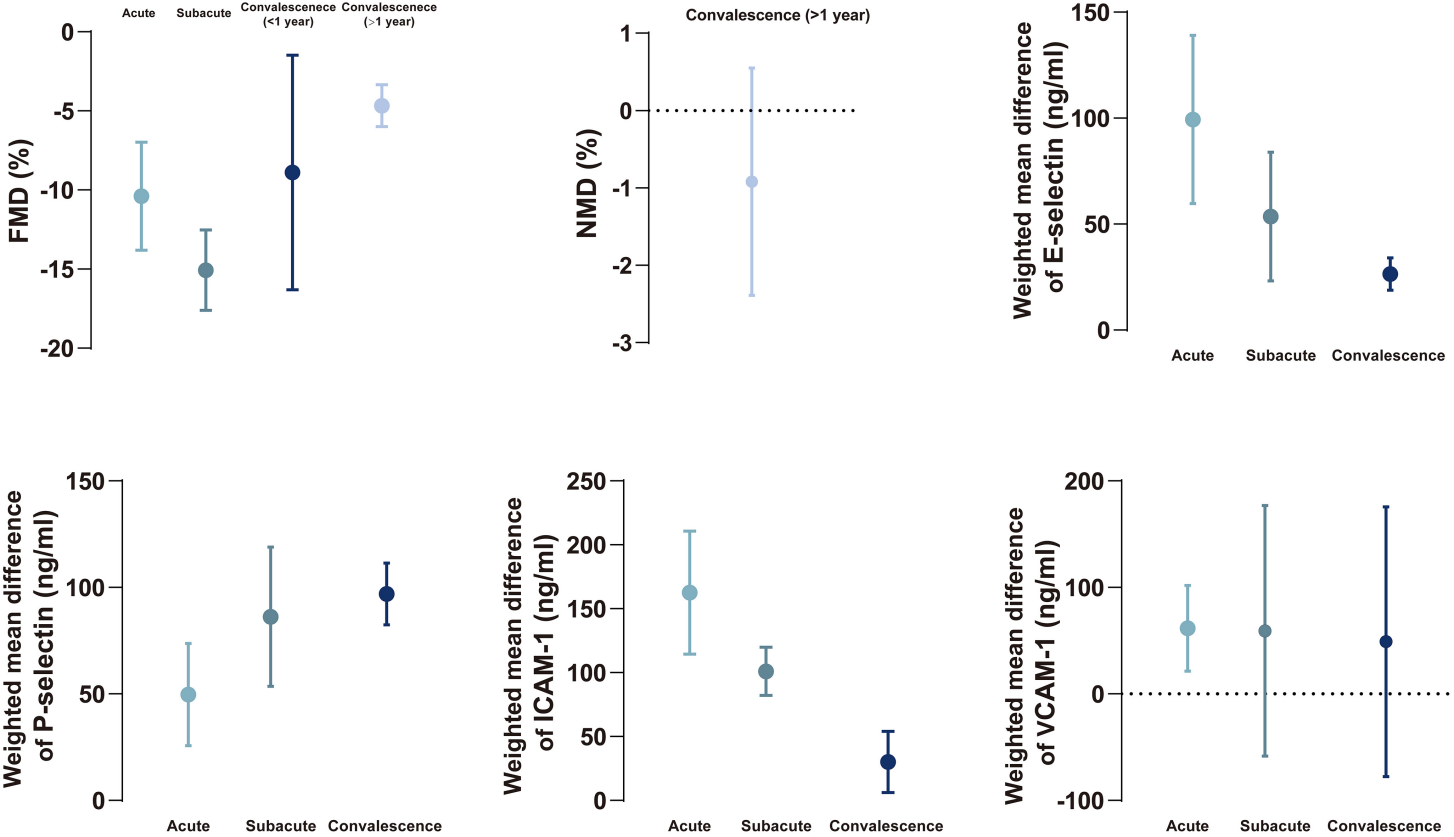
**The summary of changing tendency in FMD, NMD, biomarkers between Kawasaki disease and healthy children during different phases**. Data are expressed as mean with 95% confidence interval. FMD, flow-mediated dilatation; ICAM, intercellular adhesion molecule-1; NMD, nitroglycerin-mediated dilation; VCAM, vascular cellular adhesion molecule-1.

This is the first meta-analysis that comprehensively summarized the endothelial function alteration in KD children during different phases compared to healthy children (Fig. 5). The results were confirmed by subgroup analysis, sensitivity analysis, and publication bias test. Meanwhile, a positive correlation may exist between the degree of FMD impairment/endothelial dysfunction and the severity of CAL in KD patients during the convalescence phase.

The etiology of KD is unknown. The most reasonable hypothesis is that activation of the immune system along with the release of inflammatory cytokines, destroys the intima of the artery. Histopathological findings in acute KD show widespread vascular inflammation with endothelial edema and necrosis and leukocyte infiltration, involving coronary and other medium-sized muscular arteries [51]. Endothelial dysfunction is one of the earliest manifestations of arteriosclerosis during vascular remodeling [52]. There were several noninvasive peripheral endothelial function tests, including FMD, peripheral arterial tonometry, laser-doppler flowmetry, laser-speckle contrast imaging/analysis, and near-infrared spectroscopy [53]. The most widely used test is FMD [54].

Endothelial dysfunction is characterized by vasodilatation impairment. FMD measures changes in the endothelium-dependent vasodilator response after shear stress and the dilation induced by the release of nitric oxide [55]. FMD provides accurate prediction information for future cardiovascular events and is considered the gold standard for assessing endothelial dysfunction [54]. Several systemic reviews attempted to summarize FMD of KD during the convalescence phase [56, 57, 58, 59, 60]. They all found that FMD decreased in patients with a history of KD. However, none of those studies used subgroup analysis. Meanwhile, only Dietz and her colleagues focused on the FMD of KD patients with CAL [56]. Due to CAL can be separated into CAA group and “coronary artery dilation only” subgroup [1]. In fact, they didn’t assess the relationship between FMD and CAA status (CAL severity). In our results, FMD decreased in KD patients compared to the healthy control, regardless of whether they are in the acute, subacute, or convalescent phase. The degree of decreased FMD is phase dependent. The decrease of FMD in the subacute phase is greater than in the acute phase, and FMD decrease is greater in the subacute phase than in the convalescence phase. The reasons for this difference are not clear but may relate in part to the pathophysical changes of the arteries during different phases. In the subacute phase, luminal myofibroblastic proliferation and laminar non-occlusive thrombosis may exist, whereas in the acute phase, mild, transient dilatation may already occur [1], and in the convalescent phase (in particular, >1 year) some arterial lesions may be recovered. Additionally, the degree of FMD impairment appears to be positively correlated.

NMD, a part of the FMD protocol, is usually performed to assess endothelium-independent vasodilation and the function of vascular smooth muscle. A recent study revealed that NMD is an independent predictor of long-term cardiovascular events [61]. There was no difference in NMD between children with KD and healthy children, suggesting normal vascular smooth muscle function in patients with a history of KD and there is derangement in the vascular smooth muscle cells receptor pathway [62, 63].

E-selectin (CD62E), P-selectin (CD62P), ICAM-1 (CD54) and VCAM-1 (CD106) are cell surface adhesion molecules present on vascular endothelial cells [64, 65]. KD patients have elevated inflammatory cytokines, polyclonal B cell activation and T cell activation [66]. Those inflammatory cytokines upregulated these four biomarkers. Therefore, it has been proposed that cytokine-mediated vascular endothelial cell activation and injury is a central part of KD pathogenesis [45, 66]. In addition, oxidative stress is reported to impact vascular function by decreasing the availability of NO, leading to endothelial dysfunction [17, 41, 46]. In our results, elevated levels of E-selectin and ICAM-1 are phase dependent, with the highest levels in the acute phase and the lowest levels in the convalescent phase. The profiles of both biomarkers demonstrated that E-selectin and ICAM-1 could be used as reliable, early biomarkers for KD patients. In contrast, elevated level of P-selectin in the subacute phase is higher than that in the acute phase. The mechanism by which induced this tendence is still unknown but may be related in part to the source of release of p-selectin from both activated endothelium and activated platelets [67]. In the acute phase, the release of P-selectin may be predominately from early activated endothelial cells, whereas in the subacute phase, the release of P-selectin is derived from early activated endothelial cells, joining with the release of P-selectin by activated platelets. Therefore, P-selectin may be used as a sensitive biomarker for the subacute phase KD.

### 4.1 Future Direction

This correlation between FMD impairment degree and CAL severity needs more studies to confirm. Meanwhile, whether there is any difference in FMD between patients with persistent CAA and regressed CAA needs to be further researched. Second, decreased FMD was found in NCAL patients during the convalescence phase compared to the control group. FMD may be used as a good follow-up indicator for NCAL patients for future studies. Finally, the elevation of biomarkers of endothelial cells requires more evidence to support. The relationship between biomarkers levels and CAL severity in KD children was still unclear [68].

### 4.2 Limitation

First, single race and effects of the involved studies can’t be ignored. The scarcity of studies with FMD during the subacute phase may affect the credibility of the conclusion. Meanwhile, only a limited numbers of NMD studies were involved in our analysis. Second, all included studies had a retrospective observational design, and the data were not sufficiently matched or adjusted for confounders. Confounding factors included severity of the disease, follow-up duration, treatment in the acute phase, lifestyle, dietary habits, etc. Third, high heterogeneity was found in most analyses. Subgroup analysis was performed. No source of heterogeneity was revealed. Fourth, the publication bias was existed in some analysis, which was adjusted by the trim-and-fill method. Five, the FMD/NMD protocol in each involved study is not exactly the same.

## 5. Conclusions

Endothelial dysfunction is present since the onset of KD and persists for years, confirmed by the measurement of FMD and biomarkers from different phases. An assumption is advanced that FMD impairment may be positively correlated with CAL severity during the convalescence phase.

## Abbreviations

CAA, coronary artery aneurysm; CAL, coronary artery lesion; CI, confidence interval; FMD, flow-mediated dilatation; ICAM, intercellular adhesion molecule-1; KD, Kawasaki disease; NMD, nitroglycerin-mediated dilation; VCAM, vascular cellular adhesion molecule-1; WMD, weighted mean difference.

## Supplementary Material

Supplementary material associated with this article can be found, in the online version, at https://doi.org/10.31083/j.rcm2308260.
