# Supplementary material for: Assessment of Endothelial Dysfunction in Patients with Kawasaki Disease: A Meta-Analysis
Source: Rev Cardiovasc Med. 2022 Jul 20;23(8):260. doi: 10.31083/j.rcm2308260 (PMC11266953; doi:10.31083/j.rcm2308260)

Assessment of endothelial function in patients with Kawasaki disease: a meta-analysis

Content

Table S1 PRISMA Checklist 2009 2

Table S2 Summary quality of the studies included in this meta-analysis 4

Table S3 Details of flow-mediated dilation measurement in the involved studies..5

Table S4 Assessment of publication bias 6

Fig. S1 Evaluation of publication bias of flow-mediated dilatation in the convalescence phase 7

Fig. S2 Evaluation of publication bias of nitroglycerin-mediated dilation 7

Fig. S3 Evaluation of publication bias of four biomarkers..8

Fig. S4 Trim-and-fill analysis for publication bias of flow-mediated dilatation in the acute phase 9

Table S1 PRISMA Checklist 2009

Section/topic	#	Checklist item	Reported on page #
**TITLE**			
Title	1	Identify the report as a systematic review, meta-analysis, or both.	Title page
**ABSTRACT**			
Structured summary	2	Provide a structured summary including, as applicable: background; objectives; data sources; study eligibility criteria, participants, and interventions; study appraisal and synthesis methods; results; limitations; conclusions and implications of key findings; systematic review registration number.	1
**INTRODUCTION**			
Rationale	3	Describe the rationale for the review in the context of what is already known.	2-3
Objectives	4	Provide an explicit statement of questions being addressed with reference to participants, interventions, comparisons, outcomes, and study design (PICOS).	2-3
**METHODS**			
Protocol and registration	5	Indicate if a review protocol exists, if and where it can be accessed (e.g., Web address), and, if available, provide registration information including registration number.	3
Eligibility criteria	6	Specify study characteristics (e.g., PICOS, length of follow-up) and report characteristics (e.g., years considered, language, publication status) used as criteria for eligibility, giving rationale.	3
Information sources	7	Describe all information sources (e.g., databases with dates of coverage, contact with study authors to identify additional studies) in the search and date last searched.	3
Search	8	Present full electronic search strategy for at least one database, including any limits used, such that it could be repeated.	3
Study selection	9	State the process for selecting studies (i.e., screening, eligibility, included in systematic review, and, if applicable, included in the meta-analysis).	3-4
Data collection process	10	Describe method of data extraction from reports (e.g., piloted forms, independently, in duplicate) and any processes for obtaining and confirming data from investigators.	3-4
Data items	11	List and define all variables for which data were sought (e.g., PICOS, funding sources) and any assumptions and simplifications made.	3
Risk of bias in individual studies	12	Describe methods used for assessing risk of bias of individual studies (including specification of whether this was done at the study or outcome level), and how this information is to be used in any data synthesis.	4
Summary measures	13	State the principal summary measures (e.g., risk ratio, difference in means).	4
Synthesis of results	14	Describe the methods of handling data and combining results of studies, if done, including measures of consistency (e.g., I^2) for each meta-analysis.	4

Section/topic	#	Checklist item	Reported on page #
Risk of bias across studies	15	Specify any assessment of risk of bias that may affect the cumulative evidence (e.g., publication bias, selective reporting within studies).	4
Additional analyses	16	Describe methods of additional analyses (e.g., sensitivity or subgroup analyses, meta-regression), if done, indicating which were pre-specified.	4
**RESULTS**			
Study selection	17	Give numbers of studies screened, assessed for eligibility, and included in the review, with reasons for exclusions at each stage, ideally with a flow diagram.	4
Study characteristics	18	For each study, present characteristics for which data were extracted (e.g., study size, PICOS, follow-up period) and provide the citations.	4
Risk of bias within studies	19	Present data on risk of bias of each study and, if available, any outcome level assessment (see item 12).	6
Results of individual studies	20	For all outcomes considered (benefits or harms), present, for each study: (a) simple summary data for each intervention group (b) effect estimates and confidence intervals, ideally with a forest plot.	4-5
Synthesis of results	21	Present results of each meta-analysis done, including confidence intervals and measures of consistency.	4-5
Risk of bias across studies	22	Present results of any assessment of risk of bias across studies (see Item 15).	6
Additional analysis	23	Give results of additional analyses, if done (e.g., sensitivity or subgroup analyses, meta-regression [see Item 16]).	5
**DISCUSSION**			
Summary of evidence	24	Summarize the main findings including the strength of evidence for each main outcome; consider their relevance to key groups (e.g., healthcare providers, users, and policy makers).	6-7
Limitations	25	Discuss limitations at study and outcome level (e.g., risk of bias), and at review-level (e.g., incomplete retrieval of identified research, reporting bias).	7-8
Conclusions	26	Provide a general interpretation of the results in the context of other evidence, and implications for future research.	8
**FUNDING**			
Funding	27	Describe sources of funding for the systematic review and other support (e.g., supply of data); role of funders for the systematic review.	8

From: Moher D, Liberati A, Tetzlaff J, Altman DG, The PRISMA Group (2009). Preferred Reporting Items for Systematic Reviews and Meta-Analyses: The PRISMA Statement. PLoS Med 6(7): e1000097. doi:10.1371/journal.pmed1000097

For more information, visit: **www.prisma-statement.org**.

Table S2 Summary quality of the studies included in this meta-analysis

Study	Year	Selection				Comparability	Outcome			
		Is the case definition adequate	Representativeness of the cases	Selection of Controls	Definition of Controls		Ascertainment of exposure	Same method of ascertainment for cases and controls	Non-Response rate	Total
**Furukawa**	1992	★	★	★	★	★★	★	★		8
**Kim**	1994	★	★	★	★	★★	★	★		8
**Nash**	1995	★	★	★	★	★	★	★	★	8
**Dhillon**	1996	★	★	★	★	★	★	★		7
**Takeshita**	1997	★	★	★	★	★	★	★	★	8
**Schiller**	1999	★	★	★	★	★★	★	★		8
**Silva**	2001	★	★	★	★	★	★	★	★	8
**Deng**	2002	★	★	★	★	★★	★	★	★	9
**Qiu**	2004	★	★	★	★	★★	★	★	★	9
**Kadono**	2005	★	★	★	★	★	★	★	★	8
**Sun_1**	2005	★	★	★	★	★	★	★		7
**Sun_2**	2005	★	★	★	★	★	★			6
**Zhang**	2005	★	★	★	★	★	★	★		7
**Wang**	2006	★	★	★	★	★	★			6
**Li**	2007	★	★	★	★	★	★	★		7
**Liu**	2007	★	★	★	★	★	★	★	★	8
**McCrindle**	2007	★	★	★	★	★★	★	★	★	9
**Borzutzky**	2008	★	★	★	★	★	★	★	★	8
**Huang**	2008	★	★	★	★	★★	★	★	★	9
**Xu_1**	2008	★	★	★	★	★	★			6
**Xu_2**	2008	★	★	★	★	★	★			6
**Ghelani**	2009	★	★	★	★	★	★	★	★	8
**Liu**	2009	★	★	★	★	★★	★	★	★	9
**Chen**	2010	★	★	★	★	★★	★	★	★	9
**Straface**	2010	★	★	★	★	★	★	★	★	8
**Duan**	2011	★	★	★	★	★	★	★	★	8
**Liu**	2013	★	★	★	★	★	★	★	★	8
**Ishikawa**	2013	★	★	★	★	★★	★	★	★	9
**Ding**	2014	★	★	★	★	★	★	★	★	8
**Duan**	2014	★	★	★	★	★★	★	★	★	9
**Laurito**	2014	★	★	★	★	★	★	★	★	8
**Gao**	2015	★	★	★	★	★★	★	★	★	9
**Sabri**	2015	★	★	★	★	★	★	★		7
**Mori**	2016	★	★	★	★	★	★	★		7
**Parihar**	2017	★	★	★	★	★	★	★		7
**Ishikawa**	2018	★	★	★	★	★★	★	★	★	9
**Pi**	2018	★	★	★	★	★★	★	★		8
**Çetiner**	2021	★	★	★	★	★	★	★		7
**Routhu**	2021	★	★	★	★	★	★	★	★	8
**Wen**	2021	★	★	★	★	★★	★	★	★	9

Table S3 Details of flow-mediated dilation measurement in the involved studies

Study	Year	Occlusion position	Occlusion pressure	Occlusion duration
**Dhillon**	1996	Foream (unspecified)	300 mmHg	4.5 mins
**Silva**	2001	Foream (unspecified)	>250 mmHg	4 mins
**Deng**	2002	Foream (unspecified)	200 mmHg	5 mins
**Kadono**	2005	Foream (distal)	200 mmHg	5 mins
**Sun_1**	2005	Foream (unspecified)	50 mmHg above resting systolic blood pressure	5 mins
**Sun_1**	2005	Foream (unspecified)	50 mmHg above resting systolic blood pressure	5 mins
**Liu**	2007	Foream (unspecified)	200 mmHg	5 mins
**McCrindle**	2007	Upper arm	20 mmHg above resting systolic blood pressure	5 mins
**Borzutzky**	2008	NR	50 mmHg above resting systolic blood pressure	5 mins
**Huang**	2008	Foream (distal)	50 mmHg above resting systolic blood pressure/200 mmHg	5 mins
**Ghelani**	2009	Foream (proximal)	50 mmHg above resting systolic blood pressure	5 mins
**Liu**	2009	Foream (distal)	200 mmHg	5 mins
**Duan**	2011	Foream (unspecified)	200 mmHg	5 mins
**Ishikawa**	2013	Foream (distal)	200 mmHg	5 mins
**Ding**	2014	NR	50 mmHg above resting systolic blood pressure	5 mins
**Duan**	2014	NR	200 mmHg	5 mins
**Laurito**	2014	Foream (proximal)	250 mmHg	5 mins
**Gao**	2015	Upper arm	50 mmHg above resting systolic blood pressure	5 mins
**Sabri**	2015	Foream (distal)	NR	5 mins
**Mori**	2016	NR	250 mmHg	4–5 mins
**Parihar**	2017	NR	50 mmHg above resting systolic blood pressure	5 mins
**Ishikawa**	2018	NR	200 mmHg	5 mins
**Çetiner**	2021	Upper arm	50 mmHg above resting systolic blood pressure	5 mins
**Routhu**	2021	Foream (proximal)	250 mmHg	5 mins
**Wen**	2021	NR	50 mmHg above resting systolic blood pressure	5 mins

NR, not reported.

Table S4 Assessment of publication bias

Outcome	Test of Publication Bias Begg's *P* value	Test of Publication Bias Egger's *P* value
**FMD in the acute phase (%)**	0.221	0.038
**FMD in the subacute phase (%)**	0.999	-
**FMD in the convalescence phase (%)**	0.833	0.399
**NMD (%)**	0.902	0.630
**E-selectin (ng/ml)**	0.263	0.260
**P-selectin (ng/ml)**	0.873	0.297
**ICAM-1 (ng/ml)**	0.474	0.814
**VCAM-1 (ng/ml)**	0.536	0.135

FMD, flow-mediated dilatation; ICAM-1, intercellular adhesion molecule-1; NMD, nitroglycerin-mediated dilation; VCAM-1, vascular cellular adhesion molecule-1.

Fig. S1 Evaluation of publication bias of flow-mediated dilatation in the convalescence phase

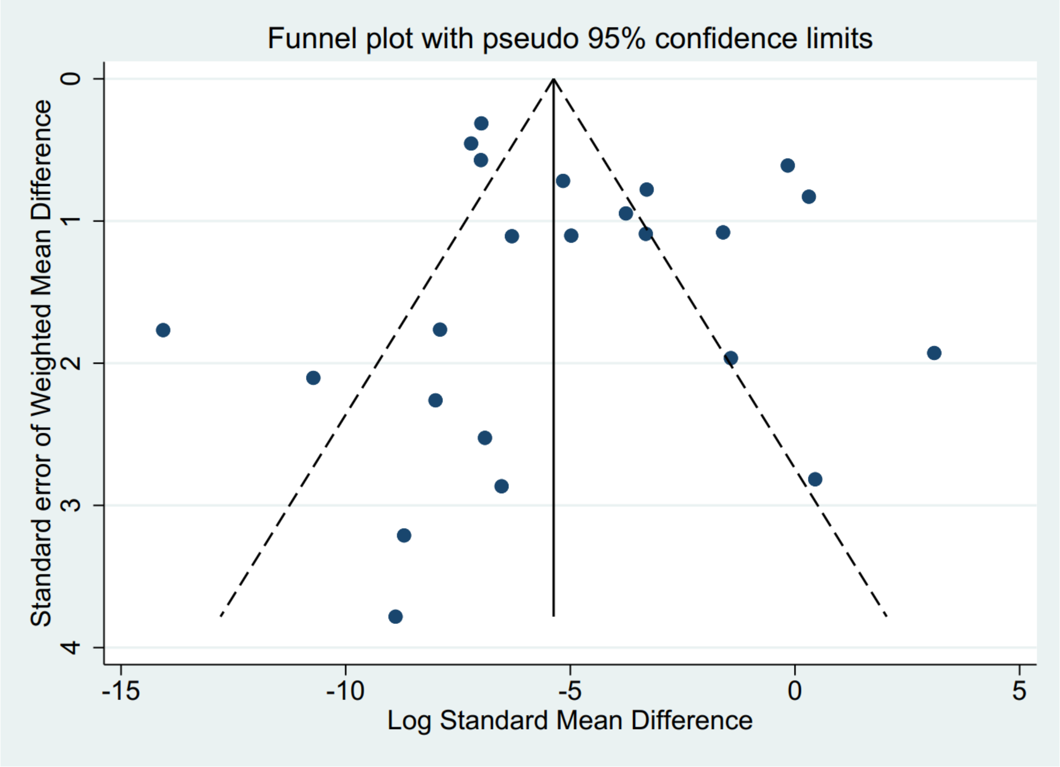

Fig. S2 Evaluation of publication bias of nitroglycerin-mediated dilation

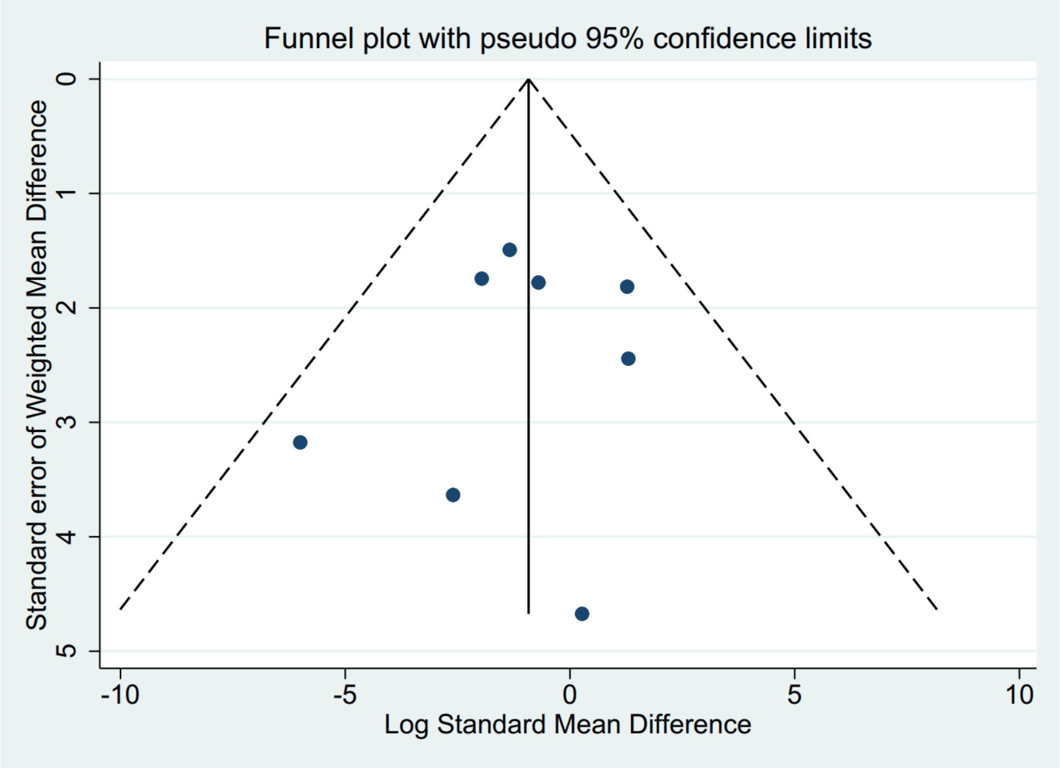

Fig. S3 Evaluation of publication bias of four biomarkers

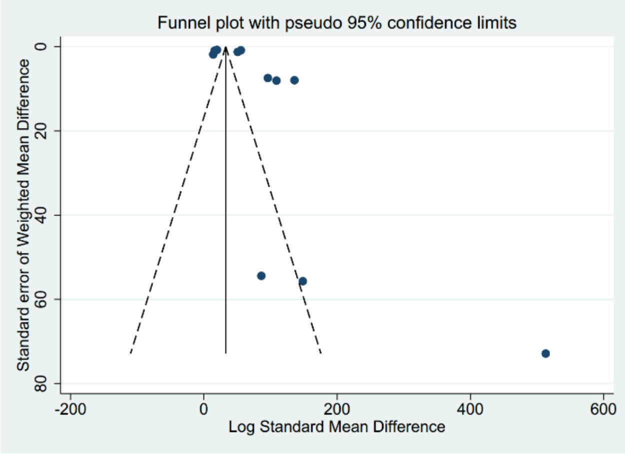

E-selectin

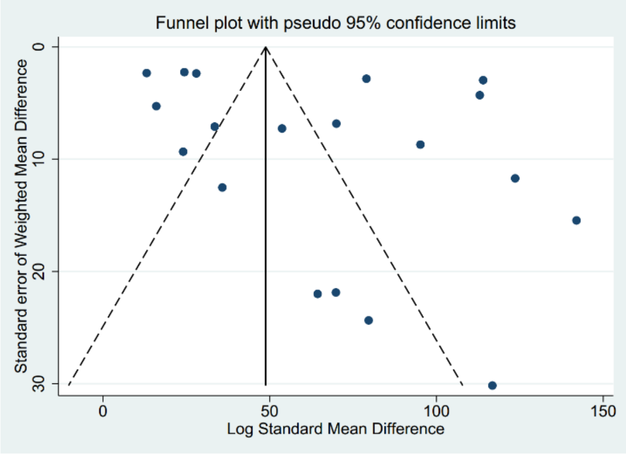

P-selectin

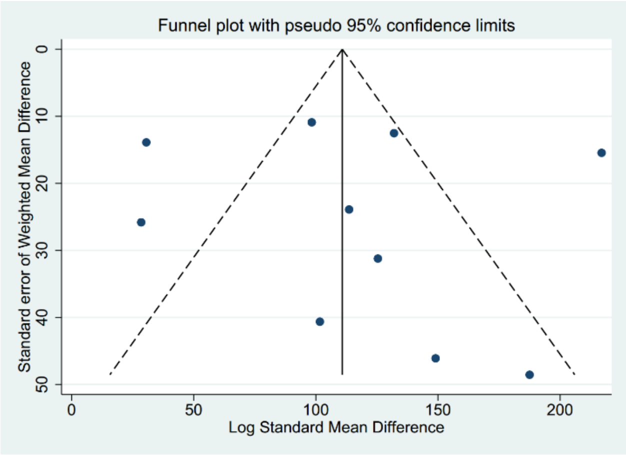

ICAM-1

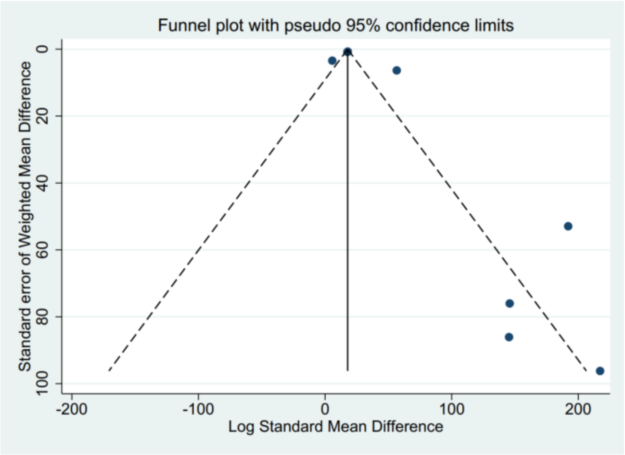

VCAM-1

Fig. S4 Trim-and-fill analysis for publication bias of flow-mediated dilatation in the acute phase

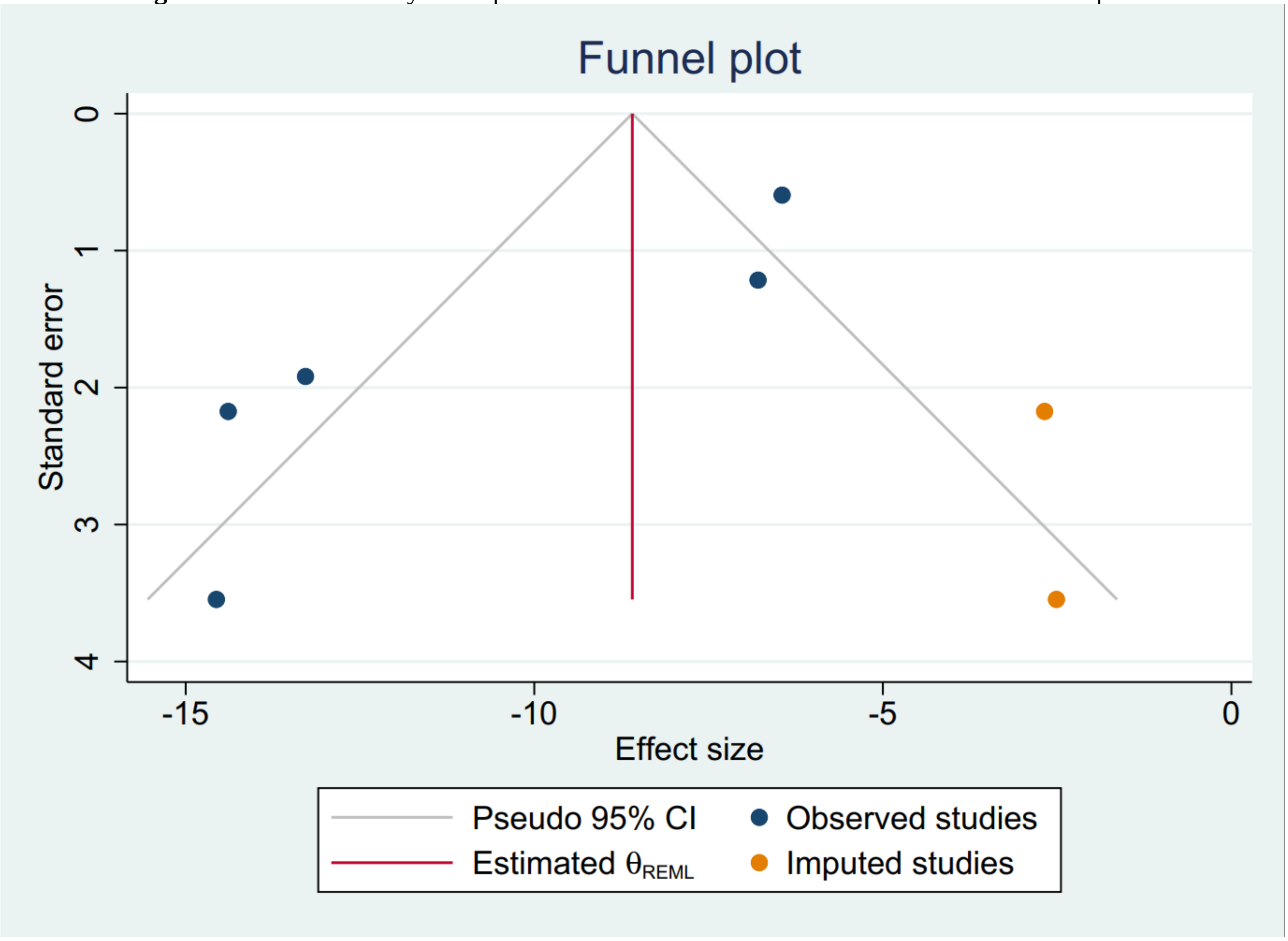

Supplement: Supplementary file 1 [file 2153-8174-23-8-260-s1.pdf]
